# Effect of educational program based on theory of planned behavior on promoting nutritional behaviors preventing Anemia in a sample of Iranian pregnant women

**DOI:** 10.1186/s12889-021-12270-x

**Published:** 2021-12-01

**Authors:** Ali Khani Jeihooni, Tayebeh Rakhshani, Pooyan Afzali Harsini, Mehdi Layeghiasl

**Affiliations:** 1 Nutrition Research Center, Department of Public Health, School of Health, Shiraz University of Medical Sciences, Shiraz, Iran; 2 Department of Public Health, School of Health, Kermanshah University of Medical Sciences, Kermanshah, Iran; 3 Department of Health Promotion, School of Health, Shiraz University of Medical Sciences, Shiraz, Iran

**Keywords:** Iron-deficiency Anemia, Students, Nutrition, Education, TPB

## Abstract

**Background:**

Iron deficiency anemia (IDA) is one of the most common problems during pregnancy. The aim of this research was to determine the effect of educational program based on Theory of Planned Behavior (TPB) on promoting nutritional behaviors preventing anemia in a pregnant woman in Shiraz city, Iran.

**Methods:**

This quasi-experimental study was done on 150 pregnant women (75 experimental and 75 control groups) who were selected using randomly sampling method in in Shiraz city, Iran, in 2020–2021. The educational intervention for the experimental group included six educational sessions for 50 or 55 min-based TPB model. A questionnaire consisted of items about demographic information, TPB constructs (attitude, perceived behavioral control, subjective norms and behavioral intention) was used to measure the nutritional behaviours preventing iron deficiency anemia in pregnancy women before and 3 months after the intervention.

**Results:**

The results showed that before the educational intervention, there was no significant difference between the two groups in terms of knowledge, attitude, perceived behavioral control, subjective norms, behavioral intention and nutritional performance; however, three months after the educational intervention, the experimental group showed a significant increase in each of the mentioned variables. For example the mean and standard deviation score of behavioral intention after intervention in the experimental group was significantly increased (25.57 ± 1.66, *P* = 0.001),and the mean and standard deviation score of performance after intervention in the experimental group was significantly increased (31.03 ± 2.19, P = 0.001), (*P* < 0.05).

**Conclusions:**

After the educational intervention, the experimental group showed a significant increase in of the knowledge, attitude, perceived behavioral control, subjective norms, behavioral intention and nutritional performance. Therefore the results of the study showed positive effect of nutrition educational intervention program base on TPB model on improvement of iron deficiency anemia preventive behaviours in the pregnancy women.

## Background

Anemia is a clinical disorder characterized by a decrease in hemoglobin concentration. Generally more than two billion people in the world, including more than half of pregnant women suffer from anemia [1, 2]. The prevalence of anemia among pregnant women in developing countries is about 40–60% [2]. The World Health Organization (WHO) also estimates the prevalence of anemia in pregnant women in the Eastern Mediterranean at 44.2%. In this report, the prevalence of anemia in Iranian pregnant women was more than 40% [3]. When the prevalence of anemia in a population reaches more than 40%, it becomes a serious health concern [4]. Therefore, iron deficiency anemia (IDA) is one of the most common problems during pregnancy [4, 5].

Iron-deficiency anemia (IDA) can cause serious harm to the mother and fetus [6]. Since pregnancy is considered as one of the most important periods in a woman’s life [7], iron deficiency anemia (IDA) has dangerous effects on mother and fetus and reduces fertility, preterm delivery, severe neonatal birth weight, increased spontaneous abortion, increased fetal mortality and postpartum hemorrhage [8, 9]. In addition, the consequences of anemia in pregnant women affect the social and economic development of individuals [10]. Anemia can be caused by iron deficiency, impaired absorption, iron loss, or increased need for iron [11].

Despite the fact that free iron supplementation has been an important part of prenatal care in primary health care centers in Iran since 1983, anemia during pregnancy is still a major nutritional problem [2]. Preventive measures aimed at controlling anemia among pregnant women are very important in terms of public health benefits and cost-effectiveness due to the reduction of mortality and prevention of its complications among mothers and infants [12]. One of the most effective ways to reduce these adverse outcomes during pregnancy is to engage in self-care behaviors [13].

The vulnerability of pregnant women and the complications of anemia make it necessary to anticipate effective and appropriate measures and programs in this regard. Recognizing behavior and the factors influencing it to change or modify existing behaviors and replace new behaviors for the effectiveness of these programs is one of the effective strategies that emphasizes the role of health education and health promotion models [8, 12].

Therefore, health education and health promotion models are good guides for analyzing the factors affecting behavior, planning, and effective intervention, and they are recommended to obtain useful and effective results [14].. The most important step in recognizing the various factors affecting behavior, in designing and implementing educational and intervention programs, is to choose the appropriate model or theory. One of the effective theories in the successful conversion of adverse behavior to healthy behavior is the theory of planned behavior (TPB) [15, 16]. This theory considers individual beliefs, social factors and motivation to follow important people in life as a network of factors influencing behavior change. As a theory of behavior change (cognitive-social model of value expectation), it states that intention is the main determinant of behavior and is influenced by the following three independent constructs:

1. An individual’s attitude toward behavior that is shaped by behavioral beliefs and evaluation of behavioral outcome.
2. The Subjective norms of this construct is shaped by normative beliefs and motivation to follow.
3. Perceived behavioral control which this construct is influenced by control belief and perceived power [15, 16].

Of course, numerous successful studies have been conducted worldwide to improve nutritional behaviors associated with iron deficiency anemia using TPB [8, 12, 17]. Therefore, considering that anemia is one of the causes of maternal mortality during pregnancy and that maternal mortality is considered as one of the important indicators of the quality of health services in a country [18] And due to nutritional problems during pregnancy, including the high prevalence of anemia in Iranian pregnant women [3] and it becomes a serious concern for their health [4]. the present study was conducted to determine the effectiveness of the TPB in promoting nutritional behaviors preventing anemia in a sample of Iranian pregnant women. Our hypothesis was that after the intervention based on “TPB”, the mean scores of nutritional behaviors preventing anemia in pregnant women were statistically significant in the experimental and control groups.

## Methods

### Study design and sample size

This quasi-experimental study was conducted on 150 pregnant women under the auspices of health centers of Shiraz in 2020–2021. For sampling, from among urban health centers of Shiraz, two centers were randomly selected (one center for the experimental group and one center for the control group). The sampling method in each health center was simple random. In this way, first a list of all pregnant mothers in each health center was prepared and then the participants were randomly selected using a random number table. The samples were then invited to attend a health center on a certain day to get acquainted with. They were also explained the study objectives, and informed consent was obtained from participants. The sample size was calculated based on Jahangiri et al. [12]. Using the formula for comparing the mean of the variables, 40 people were determined for each group. To increase the accuracy and power of the study, 75 people per group were considered. Inclusion criteria were willingness to participate and literate pregnant women at the beginning of the fourth month of pregnancy who were referred to health centers and filed a case of an underlying disease related to anemia, such as a history of inherited and acquired diseases, including thalassemia with no history of bleeding. The exclusion criteria were unwillingness to participate in the study, absence from more than two training sessions and questionnaire completion sessions, relocation of the pregnant mother and the impossibility of accessing her.

### Instruments

The data collection tool included a questionnaire that was used in the study of Jahangiri et al., whose validity and reliability were confirmed [12]. Cronbach’s alpha for knowledge, attitude, subjective norm, perceived behavioral control, behavioral intention, and performance checklist was 0.71, 0.80, 0.79, 0.76, and 0.79, respectively. The total reliability of the tool was also 0.80.

The questionnaire included demographic information (age, mother’s education, spouse’s education, occupation, gestational week, number of children, family income, and hemoglobin level) and 10 questions on knowledge. A TPB-based questionnaire includes: 12 questions on attitude (For example: like any other woman, I am at risk for complications from iron deficiency in pregnancy. I do not need to take iron pills because my nutrition is good.), measured using a five-point Likert scale from (1: I strongly agree) to (5: I strongly disagree), higher scores indicate a more positive attitude toward the corresponding health behavior. 5 questions on perceived behavioral control (For example: Even if the iron pill makes me nauseous, I still take it. Due to the high cost of meat, I can use legumes and eggs as a suitable alternative.), measured by a five-point Likert scale from (1: I strongly agree) to (5: I strongly disagree). Higher scores indicate higher levels of perceived control to engage in the corresponding health behavior. 6 questions on subjective norms (For example: My husband encourages me to eat right and take iron pills. I am encouraged by health professionals when I have the right diet and take supplements.), measured by a five-point Likert scale from (1: I strongly disagree) to (5: I strongly agree). Higher scores indicate greater perceived pressure to engage in the behavior. 6 questions on behavioral intention (For example: I plan to include iron-rich foods in my diet, such as meat and legumes. I plan to take iron pills regularly until the end of my pregnancy.), using afive-point Likert scale ranging (strongly disagree) to (strongly agree). Higher scores indicate stronger intentions to engage in the corresponding health behavior. The possible score was between − 10 to + 10 for each specific factor. and 9 questions on performance checklist.

After the selection of the experimental and control group, the purpose of the study and how to do the work were explained to the participants and the staff of the health centers. The questionnaire was completed by two experimental and control groups.

### Educational intervention

Based on the pre-test results, the educational content was prepared based on the TPB. The educational intervention for the experimental group consisted of 6 sessions of 50–55 min through lectures, questions and answers, group discussion, educational posters and pamphlets, videoclips and powerpoint. The training program was conducted by a doctor of health education and health promotion, a nutritionist and a gynecologist in collaboration with two experts from the women’s health unit of Shiraz health center. Training sessions were held for 3 groups of 25 (one session per week). These sessions were about anemia in pregnancy, infection and complications of the disease, ways to prevent infection, prevalence of anemia among pregnant women, correct or change misconceptions and strengthen true beliefs, the implementation of nutritional behaviors to prevent anemia in pregnancy and its positive effects on maternal and fetal health. Each woman was also asked to describe her previous experiences with nutrition and the behaviors they should replace. Key barriers were discussed by pregnant women themselves, and solutions were offered to overcome those barriers. The benefits of having a preventive diet and regular use of supplements and solutions to reduce gastrointestinal side effects caused by their consumption were presented to pregnant women. The nutritionist was asked to speak for 20 min about the need for anemia-preventing nutritional behaviors for pregnant women, followed by a group discussion. One of the meetings was held with the presence of spouses and officials of health centers, and they received information about nutritional behaviors preventing anemia, emphasizing their supportive role.

At the end of the sessions, women and their spouses received educational CDs and booklets. Women were divided into groups of 8–10 and groups of friends were formed. In order to maintain and promote the activity of the experimental group, they received an educational text message per week. One month and two months after the educational intervention, two virtual follow-up sessions were held for women. But the control group only received routine training at the time of referral and only face-to-face from health care providers. (The intervention plan be stated in the Table 1). Three months after the educational intervention, both experimental and control groups completed the questionnaire. The control group, at the end of the study, received an educational booklet. Five women in the experimental group and three women in the control group did not complete the post-test questionnaire; therefore, the final analysis was performed on 70 people in the experimental group and 72 people in the control group. For ethical considerations, while obtaining permission from the ethics committee of Shiraz University of Medical Sciences with the code (IR.SUMS.REC.1400.222), a written consent was obtained from participants and the goals, importance and necessity of the study was explained to them. Participants were also assured that their information would remain confidential.

**Table 1:** Intervention plan table

Session/concept	instructor, educator	Presentation methods	Educational content	Session
Knowledge and Attitude	doctor of health education and health promotion and a nutritionist and a gynecologist	lectures, questions and answers, group discussion, and PowerPoint.	Introduction anemia and introduction to Anemia status in pregnancy in Iran and the world statistics	First session
Knowledge, Attitude and Subjective Norms	doctor of health education and health promotion and a nutritionist and a gynecologist	lectures, group discussion, educational posters and pamphlets	Prevalence of anemia in pregnant women Objective complications of anemia in pregnant women	second session
Knowledge, Attitude and Subjective Norms	doctor of health education and health promotion and a nutritionist and a gynecologist	lectures, videoclips and PowerPoint.	Ways to prevent Correct or change misconceptions Strengthen true beliefs	third session
Subjective Norms, Perceived Behavioral Control	doctor of health education and health promotion and a nutritionist and a gynecologist	lectures, questions and answers, group discussion, and PowerPoint.	How to perform nutritional behaviors to prevent anemia in pregnancy and its positive effects on maternal and fetal health	fourth Session
Subjective Norms, Perceived Behavioral Control	doctor of health education and health promotion	lectures, questions and answers, group discussion, videoclips and PowerPoint.	Prevalence of anemia among pregnant women, Ways to prevent, How to perform nutritional behaviors to prevent anemia in pregnancy	fifth Session
based on all theory structures	doctor of health education and health promotion	lectures, questions and answers, group discussion, videoclips and PowerPoint.	Summary of educational texts of previous sessions based on theory structures	Sixth Session

### Analysis data

Data were analysed using SPSS 22. We conducted descriptive and inferential analyses, including frequencies (for categorical variables) and mean (for continuous variables), for all demographic data and questionnaire scores.using Chi-square, independent t-test and paired t-test and the significance level was considered 0.05.

## Results

142 pregnant women (70 in the experimental group and 72 in the control group) participated in this study. The mean and standard deviation of age in the experimental group was (31.53 ± 6.22) years and of control group was (30.49 ± 5.85) years. The mean and standard deviation of level of hemoglobin in the experimental group was (12.77 ± 1.10) and of control group was (13.09 ± 1.07). The sample housewife in the experimental group was (*N* = 60), The sample housewife in the control group was (*N* = 59), The sample employed in the experimental group was (*N* = 10), The sample employed in the control group was (*N* = 13).

Based on the independent t-test, the differences between the two groups were not significant in terms of quantitative demographic variables (age, gestational age with previous pregnancy, number of children, gestational week, hemoglobin level) (*P* < 0.05). (Table 2).

**Table 2:** Comparison of experimental and control groups in terms of quantitative demographic variables

Variable	Experimental group		Control group		*P*-value
	M	SD	M	SD	
Age	31.53	6.22	30.49	5.85	0.352
Interval between previous pregnancy	3.62	3.28	3.56	3.18	0.612
Number of children	1.22	0.65	1.08	0.61	0.655
Gestational week	17.05	1.28	16.60	1.27	0.304
Level of hemoglobin	12.77	1.10	13.09	1.07	0.376
independent t-test					

Based on the chi-square test results, in terms of qualitative demographic variables (occupation, education, spouse’s education and occupation, and monthly income), the two groups were the same (Table 3).

**Table 3:** Comparison of experimental and control groups in terms of qualitative demographic variables

Variable		Experimental group		Control group		p-value
		N	P	N	P	
Occupation	Housewife	60	85.71	59	81.94	0.464
	Employed	10	14.29	13	18.06	
Household monthly income	< 30 million Rials	36	37.14	22	30.56	0.180
	30–60 million Rials	32	45.72	30	41.66	
	>60 million Rials	12	17.14	20	27.78	
Education	Primary school	5	7.14	7	9.72	0.279
	Secondary school	16	22.86	21	29.17	
	High school	34	48.57	28	38.89	
	College	15	21.43	16	22.22	
Spouse’s education	Illiterate	4	5.71	6	8.33	0.264
	Primary school	6	8.57	8	11.11	
	Secondary school	15	21.43	18	25	
	High school	29	41.43	21	29.17	
	College	16	22.86	19	26.39	
Spouse’s occupation	Employed	38	54.29	35	48.61	0.432
	Unemployed	32	45.71	37	51.39	
Chi-square test						

The results showed that there was no significant difference between the two groups in terms of knowledge, attitude, perceived behavioral control, subjective norms, behavioral intention, and nutritional performance before the educational intervention. However, three months after the educational intervention, the experimental group showed a significant increase in each of the mentioned variables.

According to the paired t-test, the mean and standard deviation score of knowledge after intervention in the experimental group was significantly increased (8.19 ± 1.05, *P* = 0.001), while the mean and standard deviation score of knowledge after intervention in the control group was not significant (4.39 ± 1.14, *P* = 0.262). In addition, after the intervention, the mean and standard deviation score of attitude after intervention in the experimental group was significantly increased (50.24 ± 4.28, *P* = 0.001), the mean and standard deviation score of subjective Norms after intervention in the experimental group was significantly increased (23.10 ± 1.70, *P* = 0.001), the mean and standard deviation score of perceived behavioral control after intervention in the experimental group was significantly increased (20.88 ± 1.64, *P* = 0.001), the mean and standard deviation score of behavioral intention after intervention in the experimental group was significantly increased (25.57 ± 1.66, P = 0.001),and the mean and standard deviation score of performance after intervention in the experimental group was significantly increased (31.03 ± 2.19, P = 0.001), but these factors did not change significantly after intervention in the control group (Table 4).

**Table 4:** Comparison of mean scores of knowledge, attitude, perceived behavioral control, subjective norms, behavioral intention and nutritional performance of pregnant women in experimental and control groups before and three months after educational intervention

Variable	group	Before intervention M ± SD	After intervention M ± SD	P-value
Knowledge	Experimental	4.14 ± 1.25	8.19 ± 1.05	0.001
	Control	4.25 ± 1.08	4.39 ± 1.14	0.262
	The significance level	0.258	0.001	
Attitude	Experimental	26.14 ± 4.55	50.24 ± 4.28	0.001
	Control	28.50 ± 4.16	29.18 ± 4.22	0.233
	The significance level	0.239	0.001	
Perceived behavioral control	Experimental	10.26 ± 1.73	20.88 ± 1.64	0.001
	Control	10.15 ± 1.66	11.02 ± 1.68	0.237
	The significance level	0.287	0.001	
Subjective Norms	Experimental	13.18 ± 1.92	23.10 ± 1.70	0.001
	Control	12.58 ± 1.89	13.35 ± 1.78	0.253
	The significance level	0.241	0.001	
Behavioral intention	Experimental	15.86 ± 1.40	25.57 ± 1.66	0.001
	Control	14.17 ± 1.62	16.02 ± 1.58	0.214
	The significance level	0.224	0.001	
Performance	Experimental	17.05 ± 2.21	31.03 ± 2.19	0.001
	Control	18.06 ± 2.27	19.26 ± 2.34	0.251
	The significance level	0.245	0.001	
paired t-test				

## Discussion

The present study aimed to investigate the effect of educational intervention based on the TPB on promoting nutritional behaviors preventing anemia in a sample of Iranian pregnant women. There are several risks to mothers during pregnancy. Anemia is one of the most common blood disorders and risks during pregnancy that may affect the health of mother and fetus; therefore, identifying the factors affecting the improvement of nutritional behaviors preventing anemia in pregnant women is of great importance.

In the present study, the mean score of knowledge, attitude, subjective norms, perceived behavioral control, behavioral intention and nutritional performance in both experimental and control groups before the intervention were not statistically significant. This is consistent with the results of some other studies [8, 17, 19, 20]. Perhaps the low mean score of these constructs before the educational intervention is due to the lack of codified and model-oriented training programs using participatory programs and combined educational methods [21].

However, three months after the intervention program, the mean score of knowledge, attitude, subjective norms, perceived behavioral control, behavioral intention and nutritional performance in the experimental group were statistically significant with that of the control group. This was consistent with the results of other studies, indicating the effectiveness of TPB-based educational intervention [8, 22]. Studies by Kamalifard, M. et al. [21], Anderson, A. S. et al. [23] concluded that the training package in their study could improve the nutritional knowledge, attitudes and behaviors of pregnant mothers.

The mean score of knowledge in the present study in the experimental group increased significantly after the intervention. This, consistent with other studies, showed that to improve nutritional behaviors during pregnancy, increasing mothers’ knowledge is a necessary and effective [24, 25]. Of course, the willingness of the participants to participate in the training programs is very important; for example, the results of a study by Jarrah S. S. et al. showed that most participants are very aware of the prevention of anemia and iron deficiency, but many of them wanted to know more [26].

A study by Pawlak R. et al. showed that applying this theory could lead to a positive attitude towards behavioral intention and improve nutritional behaviors [27]. In this regard, it can be concluded that in the present study, educational intervention based on the TPB could be effective in improving the attitude of pregnant mothers. Consistent with our study, the results of other studies indicated that educational programs have an effective role in creating a positive attitude toward nutritional behaviors [8, 28].

Subjective norms are considered as a secondary predictor of behavioral intention, and a person who believes that certain people approve of behavior and are motivated to meet their expectations has positive subjective norms. In this study, spouses and officials of health centers were considered as subjective norms. Similar studies have also considered the role of spouses and friends in subjective norms [8, 29]. Based on Table 4, the mean score of subjective norms in the experimental group was significantly different after the educational intervention, which is consistent with the results of some other studies [17, 19]. However, this is not consistent with the results of studies by Ahmadi et al. [30] and Pawlak et al. [27]. This could have a variety of reasons, including cultural differences and even people who are considered important in the study. For example, in their study, Jarrah SS et al. Discuss the role of cultural conditions in transmitting important health information to others.

Perceived behavioral control refers to an individuals’ understanding of how much control they have over their voluntary actions. Our intervention program in the present study could improve the perceived behavioral control in the experimental group. The results of this study were consistent with the results of the study by Alami et al. [17]. Of course, several studies have examined this issue from different aspects. Lack of financial resources, inadequate access to health care centers are among the concerns associated with reducing healthy nutritional behaviors [26, 31]. Or, for example, another study states that if pregnant women feel that environmental factors such as facilities and barriers are under their control, they will have desirable nutritional behaviors [27].

Based on the TPB, the most important determinant of behavior is behavioral intention. In this study, behavioral intention also showed a significant increase in the experimental group three months after the educational intervention. This is consistent with the results of other similar studies [8, 17, 19].

The mean score of nutritional performance in the experimental group showed a significant increase after the educational intervention, while no significant change was observed in the control group. The results of this study showed that the training program was effective in promoting optimal nutritional behaviors and was consistent with the results of other similar studies [19, 32]. Also, Jalambadani et al. could significantly improve the nutritional behaviors associated with folic acid consumption in pregnant women after the intervention [22].

The results of this study showed that implementing intervention programs using theory and model of health education and health promotion can effectively improve the desired preventive behaviors. The results of numerous similar studies that have used the TPB support the results of the present study [8, 17, 22].

The present study also had some limitations. Household economic status was one of the variables there search team was forced to disregard this variable because of the participants’ lack of clear responses to the issue, and the lack of accurate information on their economic status in the household file.

## Conclusion

Carefully choosing appropriate theories of health education and health promotion in the design and implementation of educational interventions is one of the most important factors in the effectiveness of educational interventions. Using these theories, the most important components and determinants of health behavior are identified and the components of educational programs could improve the desired and effective components at a higher cost, increasing the quality and effectiveness of educational programs. In this study, the TPB-based educational intervention could improve the nutritional behaviors of pregnant women; therefore, our suggestion is that the theory could be used as a suitable framework in a wider population to improve the nutritional performance associated with iron deficiency anemia in pregnant women.

## Data Availability

The datasets used and/or analysed during the current study are available from the corresponding author upon reasonable request.

## Abbreviations

IDA: Iron Deficiency Anemia
TPB: Theory of Planned Behavior.
